# Molecular mechanisms underlying the antidepressant actions of arketamine: beyond the NMDA receptor

**DOI:** 10.1038/s41380-021-01121-1

**Published:** 2021-05-07

**Authors:** Yan Wei, Lijia Chang, Kenji Hashimoto

**Affiliations:** 1 Division of Clinical Neuroscience, Chiba University Center for Forensic Mental Health, Chiba, Japan; 2 Key Laboratory of Medical Electrophysiology of Ministry of Education and Medical Electrophysiological Key Laboratory of Sichuan Province, Collaborative Innovation Center for Prevention and Treatment of Cardiovascular Disease, Institute of Cardiovascular Research, Southwest Medical University, Luzhou, Sichuan China

**Keywords:** Neuroscience, Depression

## Abstract

The discovery of robust antidepressant actions exerted by the *N*-methyl-D-aspartate receptor (NMDAR) antagonist (*R,S*)-ketamine has been a crucial breakthrough in mood disorder research. (*R,S*)-ketamine is a racemic mixture of equal amounts of (*R*)-ketamine (arketamine) and (*S*)-ketamine (esketamine). In 2019, an esketamine nasal spray from Johnson & Johnson was approved in the United States of America and Europe for treatment-resistant depression. However, an increasing number of preclinical studies show that arketamine has greater potency and longer-lasting antidepressant-like effects than esketamine in rodents, despite the lower binding affinity of arketamine for the NMDAR. In clinical trials, non-ketamine NMDAR-related compounds did not exhibit ketamine-like robust antidepressant actions in patients with depression, despite these compounds showing antidepressant-like effects in rodents. Thus, the rodent data do not necessarily translate to humans due to the complexity of human psychiatric disorders. Collectively, the available studies indicate that it is unlikely that NMDAR plays a major role in the antidepressant action of (*R,S*)-ketamine and its enantiomers, although the precise molecular mechanisms underlying antidepressant actions of (*R,S*)-ketamine and its enantiomers remain unclear. In this paper, we review recent findings on the molecular mechanisms underlying the antidepressant actions of (*R,S*)-ketamine and its potent enantiomer arketamine. Furthermore, we discuss the possible role of the brain–gut–microbiota axis and brain–spleen axis in stress-related psychiatric disorders and in the antidepressant-like action of arketamine. Finally, we discuss the potential of arketamine as a treatment for cognitive impairment in psychiatric disorders, Parkinson’s disease, osteoporosis, inflammatory bowel diseases, and stroke.

## Introduction

Depression is a common psychiatric disorder affecting more than 264 million people from teenagers through to older adults [1, 2]. The World Health Organization reports that ~800,000 people die from suicide every year, indicating a serious global public health problem. Delayed effects (lag time) and treatment failure through high nonresponse rates are the disadvantages of existing antidepressants such as selective serotonin-reuptake inhibitors and serotonin norepinephrine-reuptake inhibitors [3, 4]. Therefore, the development of rapid-acting antidepressants that are also effective in treatment-resistant depression is of great importance.

Abnormalities in glutamatergic neurotransmission via the *N*-methyl-D-aspartate receptor (NMDAR) play a role in the pathogenesis of mood disorders, including major depressive disorder (MDD) and bipolar disorder (BD) [5–15]. The serendipitous discovery of robust antidepressant effects exerted by the NMDAR antagonist (*R*,*S*)-ketamine was a paradigm shift in the research of mood disorders [16–18]. In 2000, Berman et al. [19] demonstrated that a single intravenous infusion of (*R*,*S*)-ketamine (0.5 mg/kg) acted within hours to cause rapid antidepressant effects in patients (*n* = 7) with MDD that were sustained for up to 72 h after treatment. Many subsequent studies showed that a single or repeated intravenous infusions of (*R*,*S*)-ketamine (0.5 mg/kg) produced robust antidepressant and antisuicidal effects in patients with treatment-resistant MDD or BD [20–28]. Furthermore, beneficial effects of (*R*,*S*)-ketamine were also exhibited in patients with treatment-resistant post-traumatic stress disorder (PTSD) [29, 30]. Despite the lack of long-term data on efficacy and limited data on safety, off-label use of (*R*,*S*)-ketamine is increasing in the United States of America (USA) and Europe [31–33].

In 2019, a Johnson & Johnson nasal spray containing the ketamine enantiomer (*S*)-ketamine (esketamine) was approved in the USA and Europe for treatment-resistant depression [34, 35], although several concerns about the efficacy and the approval were raised [36, 37]. We have proposed that the alternative ketamine enantiomer, (*R*)-ketamine (arketamine), may be a safer antidepressant than (*R*,*S*)-ketamine and esketamine [38–44].

The molecular mechanisms underlying the antidepressant actions of (*R*,*S*)-ketamine remain poorly understood. Previously, we published review articles on the mechanisms underlying the antidepressant action of ketamine and its enantiomers [41–44]. Since these reviews were published, new studies have shed further light on the molecular mechanisms of ketamine’s antidepressant effects. Here we review the recent findings on molecular mechanisms underlying the antidepressant actions of (*R*,*S*)-ketamine and its enantiomers. We also discuss the possible role of the brain–gut–microbiota and brain–spleen axes in stress-related psychiatric disorders and in the antidepressant-like action of arketamine. Finally, we discuss the potential of arketamine for the treatment of nonpsychiatric disorders.

### Beyond NMDAR inhibition

#### Preclinical findings using two enantiomers

**Fig. 1:**
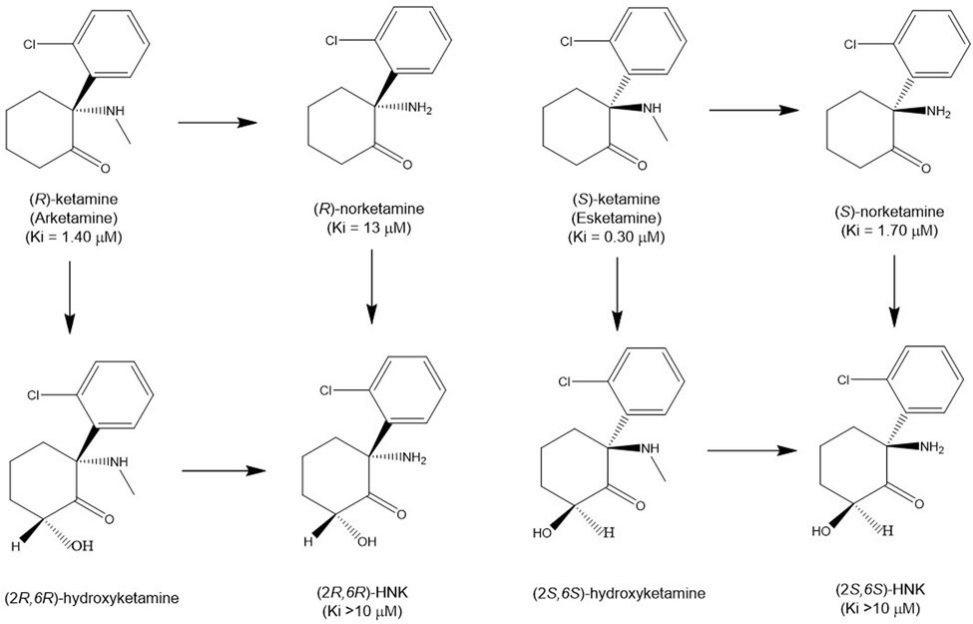
Major metabolisms of ketamine enantiomers. Arketamine is metabolized to (*R*)-norketamine that is metabolized to (2 *R*,6 *R*)-hydroxynorketamine (HNK). In addition, arketamine is also metabolized to (2 *R*,6 *R*)-hydroxyketamine that is metabolized (2 *R*,6 *R*)-HNK. Esketamine is metabolized to (*S*)-norketamine that is metabolized to (2 *S*,6 *S*)-HNK. In addition, esketamine is metabolized to (2 *S*,6 *S*)-hydroxyketamine that is metabolized to (2 *S*,6 *S*)-HNK. Ki values for NMDAR are presented in parenthesis [46, 49].

In 1975, the first study showing antidepressant-like effects of (*R,S*)-ketamine in rodents was published, reporting an amelioration of phenotype in classic animal models such as tetrabenazine-induced ptosis, reserpine-induced hypothermia, yohimbine toxicity, and oxotremorine-induced tremors [45]. (*R*,*S*)-ketamine (inhibition constant Ki = 0.53 μM for NMDAR) is a mixture of equal amounts of arketamine (Ki = 1.4 µM for NMDAR) and esketamine (Ki = 0.30 μM for NMDAR) (Fig. 1) [46]. We previously reported that arketamine exhibited greater potency and longer-lasting antidepressant-like effects than esketamine in the neonatal dexamethasone exposure, chronic social defeat stress (CSDS), and learned helplessness (LH) rodent models of depression [47, 48]. The superior effect of arketamine compared to esketamine in rodents was subsequently replicated [49–52]. Furthermore, arketamine acted with greater potency than esketamine for depression-like behaviors in an organophosphate-exposed rat model of Gulf War illness [53].

Behavioral abnormalities in rodents (hyperlocomotion, prepulse inhibition, and abuse liability) were induced less by arketamine than by esketamine [48, 52]. Furthermore, repeated intermittent administration of esketamine, but not arketamine, in mice caused a reduction in parvalbumin (PV) immunoreactivity in the prefrontal cortex (PFC) [54] and an increase in locomotion after methamphetamine administration [55]. These findings suggest a risk for psychosis in subjects administered repeated doses of esketamine. A single intravenous injection of esketamine (0.5 mg/kg), but not arketamine (0.5 mg/kg), reduced the binding availability of the dopamine D_2/3_ receptor in the conscious monkey striatum [56], suggesting that esketamine, but not arketamine, can cause dopamine release in the striatum. Thus, it has been suggested that esketamine-induced dopamine release from presynaptic terminals is associated with the psychotomimetic and dissociative effects of esketamine in humans. Collectively, data from preclinical studies suggest that arketamine elicits fewer side effects than esketamine. Indeed, it was suggested that the side effects of (*R,S*)-ketamine are associated with esketamine but not arketamine [57].

Masaki et al. [58] used functional MRI (fMRI) to investigate the response of the conscious rat brain to (*R,S*)-ketamine, its two constituent enantiomers, and the potent selective NMDAR antagonist (+)-MK-801. The positive fMRI response observed in the rat brain following a single injection of (*R,S*)-ketamine (10 mg/kg) or esketamine (10 mg/kg) was similar to the positive response observed following (+)-MK-801 (0.1 mg/kg) injection. In contrast, a single injection of arketamine (10 mg/kg) in the rat brain elicited a negative fMRI response. Thus, it is possible that the pharmacological action of 10 mg/kg arketamine, a dose capable of exerting antidepressant effects, is not dependent upon NMDAR inhibition [58]. Furthermore, other mechanisms to counteract the NMDAR inhibition-induced brain activation may exist in the negative fMRI response to arketamine; however, further study in this area is needed. The findings of Masaki et al. [58] were supported by data showing the arketamine- and esketamine-induced opposite patterns of cerebral glucose utilization in the healthy human brain [59]. Importantly, the pharmacokinetic profile of the two ketamine enantiomers was similar [49, 50], indicating that pharmacokinetic differences do not underlie the difference in the potency of the two ketamine enantiomers [41, 44]. Furthermore, (*R,S*)-ketamine was reported to exert NMDAR-independent, cAMP-dependent antidepressant-like actions [60]. Taken together, these data indicate it is unlikely that NMDAR inhibition plays a major role in the antidepressant-like actions of arketamine in rodents.

#### Preclinical findings using ketamine metabolites

Ketamine is metabolized to the intermediate norketamine (its major metabolite, 80%) via hydroxylation, or can also be metabolized to 4- and 5-hydroxyketamine (5%). Norketamine is converted to 4-, 5-, and 6-hydroxynorketamine (HNK) (15%), making HNKs and hydroxyketamines minor metabolites [44, 61–63]. In 2016, Zanos et al. [49] demonstrated that (2 *R*,6 *R*)-HNK (Ki > 10 μM for NMDAR) (Fig. 1) derived from arketamine was necessary for the antidepressant-like effects of (*R,S*)-ketamine, and that NMDAR inhibition does not play a role in the antidepressant-like effects of (2 *R*,6 *R*)-HNK since it does not bind to NMDAR [49]. In contrast, we argued that (2 *R*,6 *R*)-HNK, unlike its parent compound arketamine, did not show potent antidepressant-like effects in rodent models of depression, including CSDS and LH models [64–67]. Treatment with two cytochrome P450 (CYP) inhibitors, ticlopidine hydrochloride and 1-aminobenzotriazole, prior to arketamine administration, increased the plasma levels of arketamine, but almost completely blocked generation of (2 *R*,6 *R*)-HNK. It should be noted that the dose at which arketamine produced antidepressant-like effects was lower in the presence of CYP inhibitors than in their absence, which is consistent with antidepressant-like effects being dependent upon exposure levels of arketamine but not (2 *R*,6 *R*)-HNK [68]. Importantly, (*R*)-norketamine is a major metabolite of arketamine, while (2 *R*,6 *R*)-HNK is a minor metabolite [68]. If (2 *R*,6 *R*)-HNK is responsible for the antidepressant-like effects of (*R,S*)-ketamine, higher doses of (2 *R*,6 *R*)-HNK are needed to cause antidepressant-like effects in rodents [44].

The direct infusion of arketamine into the medial PFC and hippocampus caused antidepressant-like effects in a rat LH model, suggesting that arketamine itself, but not its metabolites, is responsible for causing antidepressant-like effects [69]. Furthermore, (2 *R*,6 *R*)-HNK (10 or 20 mg/kg) did not cause antidepressant-like effects in the repeated corticosterone injection model [70]. From the relationship between antidepressant-like effects and brain concentrations of (2 *R*,6 *R*)-HNK, Chaki and Yamaguchi [71] conclude that generation of (2 *R*,6 *R*)-HNK is not necessary for the antidepressant action of (*R,S*)-ketamine. Taken together, these data indicate that the generation of (2 *R*,6 *R*)-HNK from arketamine is not essential for the antidepressant-like effects of (*R,S*)-ketamine or arketamine [41–44, 71–74].

Antidepressant-like effects of (2 *R*,6 *R*)-HNK in chronic corticosterone-treated model, CSDS model, chronic restraint stress model, and modified LH model were demonstrated [49, 75]. In contrast, several groups reported antidepressant-like effects of (2 *R*,6 *R*)-HNK in naive rodents without a depression-like phenotype, although the effects of the parent compound arketamine were not compared in the same model [76, 77]. Importantly, in healthy control subjects, (*R,S*)-ketamine caused depressive symptoms such as anhedonia rather than antidepressant effects [78]. Thus, the use of rodents without depression-like phenotypes may lead to misinterpretation of the antidepressant-like effects of (*R,S*)-ketamine and ketamine-like candidates [79].

Esketamine is metabolized to (*S*)-norketamine (Ki = 1.07 μM for NMDAR) (Fig. 1) [46] and subsequently converted to (2 *S*,6 *S*)-HNK (Fig. 1). We reported that (*S*)-norketamine produced rapid and sustained antidepressant-like effects in mouse models of depression, with a potency similar to that of esketamine [80]. However, the antidepressant-like effects of (*S*)-norketamine were still less potent than those of arketamine. Antidepressant-like effects of (*S*)-norketamine were also reported in the repeated corticosterone-treated model [70]. (*S*)-norketamine did not cause the behavioral and biochemical abnormalities such as prepulse inhibition deficits, reward effects, loss of PV immunoreactivity in the PFC, and baseline γ-band oscillation increase that have been observed with esketamine [80]. Esketamine shares the serious detrimental side effects of (*R*,*S*)-ketamine, such as psychotomimetic and dissociative effects as well as abuse liability. These data support that NMDAR may not play a major role in antidepressant-like effects of (S)-norketamine, while it may be related to side effects. Collectively, these data suggest that (*S*)-norketamine would be a safer antidepressant than esketamine [81]. In addition, it is noteworthy that (*S*)-norketamine is not a scheduled compound and could be brought by depressed patients to their home.

#### Clinical findings

It has been recognized that a major pharmacological effect of (*R*,*S*)-ketamine is NMDAR antagonism, and that this inhibition plays a major role in the antidepressant action of (*R*,*S*)-ketamine [16, 17]. Several pharmaceutical companies developed novel NMDAR antagonists and modulators to trial as antidepressant candidates without the side effects of ketamine. However, the non-ketamine NMDAR ligands memantine, rapastinel, lanicemine, traxoprodil, and L-4-chlorokynurenine (AV-101) did not mimic the robust antidepressant actions of ketamine in patients with MDD [24, 25, 41–44, 82]. Importantly, it is well known that the potent selective NMDAR antagonist (+)-MK-801 did not cause antidepressant actions in patients with MDD [unpublished data of Merck, 44], although (+)-MK-801 had rapid antidepressant-like effects in a preclinical CSDS model [83]. Thus, it seems that the use of behavioral tests in rodent models of depression may not predict antidepressant effects in depressed patients, as the full complexity of human depression cannot be appropriately mimicked in rodents [84].

A recent meta-analysis concluded that intravenous (*R,S*)-ketamine appears more efficacious than intranasal esketamine for the treatment of depression [85], although the route of administration is different. A head-to-head study of intravenous esketamine (0.25 mg/kg) and (*R*,*S*)-ketamine (0.5 mg/kg) in Brazil found that both compounds exerted similar antidepressant effects in patients with treatment-resistant MDD; however, the sustained antidepressant effects of (*R*,*S*)-ketamine were more potent than those of esketamine at seven days after injection, albeit without statistical significance (*P* = 0.08) [86]. Furthermore, an open-label study in Brazil demonstrated that a single intravenous infusion of arketamine (0.5 mg/kg) caused rapid and sustained antidepressant effects in patients with treatment-resistant MDD, although sample size (*n* = 7) was small [87]. A recent randomized, placebo-controlled, crossover study of (*R,S*)-ketamine (0.5 mg/kg) in patients with treatment-resistant MDD showed an inverse relationship between (2 *R*,6 *R*;2 *S*,6 *S*)-HNK concentration and antidepressant response [88], suggesting that (2 *R*,6 *R*)-HNK might not contribute to the antidepressant action of (*R,S*)-ketamine [89]. A phase 1 study of (2 *R*,6 *R*)-HNK in healthy control subjects (0.1–4.0 mg/kg, intravenous administration for 40 min, NCT04711005) is underway at Duke University (sponsored by the National Institute of Mental Health, USA). A future double-blind, randomized study of arketamine versus (2 *R*,6 *R*)-HNK in MDD patients will be of great interest to confirm whether it is arketamine itself, or its metabolite, which contributes to antidepressant actions.

Taken together, it is unlikely that NMDAR inhibition plays a major role in the antidepressant effects of (*R*,*S*)-ketamine and its enantiomers in MDD patients [41–44, 90]. Nonetheless, a double-blind, randomized study of arketamine versus esketamine in MDD patients would be of great interest in confirming the role of NMDAR in the antidepressant action of (*R*,*S*)-ketamine.

#### Ketamine-induced dissociation in humans

NMDAR antagonists such as (*R,S*)-ketamine and phencyclidine (PCP) are well known to produce dissociation, such as an “out-of-body experience”, in humans [91, 92]. It was previously suggested that dissociative symptoms caused by (*R,S*)-ketamine infusion might be associated with the antidepressant action in MDD patients [93]. However, Ballard and Zarate [94] recently reported that dissociation is not necessary for the antidepressant response to (*R,S*)-ketamine and a recent systematic review showed that the association between (*R,S*)-ketamine-induced dissociation and its antidepressant effects was inconsistent [95]. Intravenous administration of esketamine (0.2 and 0.4 mg/kg) was also reported to produce dissociation in patients with treatment-resistant MDD [96]; in contrast, the incidence of dissociation after intravenous arketamine (0.5 mg/kg) administration in patients with treatment-resistant MDD is very low [87]. It is well accepted that NMDAR antagonists such as ketamine and PCP cause detrimental side effects, including psychosis and dissociation in humans in proportion to their potency at the NMDAR [97], and the difference in NMDAR potency between the two ketamine enantiomers may explain the divergence in their dissociative potential. Collectively, these studies indicate that it is unlikely that NMDAR plays a major role in the antidepressant actions of (*R,S*)-ketamine. Nonetheless, it will be of great interest to compare the antidepressant and dissociative effects of arketamine and esketamine in MDD patients [98].

### AMPAR activation

#### Ketamine and its two enantiomers

It has been recognized that the rapid-acting antidepressant-like effects of (*R,S*)-ketamine are mediated through blockade of NMDARs located on γ-aminobutyric acid (GABA)ergic inhibitory interneurons, and that subsequent activation of the α-amino-3-hydroxy-5-methyl-4-isoxazolepropionic acid receptor (AMPAR) is required for ketamine’s antidepressant effects [99, 100]. Preclinical studies showed that pretreatment with the AMPAR antagonist 2,3-dihydroxy-6-nitro-7-sulfamoylbenzo(F)quinoxaline (NBQX) could block the acute and sustained antidepressant-like effects of (*R*,*S*)-ketamine [101–103], and post treatment with NBQX reversed the effects of (*R*,*S*)-ketamine [104]. Furthermore, we reported that NBQX blocked the acute and sustained antidepressant-like effects of both arketamine and esketamine in a CSDS model [48]. Therefore, it appears likely that AMPAR activation may be necessary for rapid and sustained antidepressant-like effects of (*R*,*S*)-ketamine and its two constituent enantiomers [41–44, 105]. In order to confirm the role of AMPAR in the antidepressant actions of (*R,S*)-ketamine in humans, two clinical trials using AMPAR antagonist perampanel (Fycompa^Ⓡ^) in patients with treatment-resistant MDD are underway at Yale University (NCT03367533) and the National Institute of Mental Health (NCT03973268).

#### (*S*)-norketamine

In contrast, we reported that AMPAR antagonists NBQX or 6-cyano-7-nitroquinoxaline-2,3-dione (CNQX) did not block the rapid-acting antidepressant-like effects of (*S*)-norketamine, the major metabolite of esketamine, in a CSDS model, nor did (*S*)-norketamine enhance AMPAR-mediated neurotransmission in hippocampal neurons [80]. Our data suggest that the metabolite (*S*)-norketamine exerts AMPAR-activation-independent antidepressant-like actions in rodents. Further study on the role of AMPAR activation in the antidepressant effects of (*R,S*)-ketamine and its metabolites is needed.

### Brain-derived neurotrophic factor and its receptor TrkB system

#### BDNF–TrkB system

Multiple lines of evidence suggest that brain-derived neurotrophic factor (BDNF) and its receptor tropomyosin receptor kinase B (TrkB) play a crucial role in depression and in the therapeutic mechanisms of antidepressants [106–112]. In 2002, Shirayama et al. [113] reported in a rat LH model that a single bilateral infusion of BDNF into the hippocampal dentate gyrus produced rapid antidepressant-like effects that were sustained for at least 10 days, indicating a key role for BDNF in antidepressant mechanisms. Garcia et al. [114] reported that ketamine (10 and 15 mg/kg) increased hippocampal BDNF, which may contribute to the antidepressant-like effect of BDNF in rats. However, neither acute nor chronic administration of ketamine altered hippocampal BDNF levels in rats exposed to stress [115].

In 2011, Autry et al. [116] demonstrated that (*R,S*)-ketamine did not cause antidepressant-like effects in inducible *Bdnf* knockout (KO) mice, suggesting that the rapid synthesis of BDNF is necessary for the antidepressant-like effects of (*R,S*)-ketamine. Pretreatment with the TrkB inhibitor ANA-12 in a CSDS model blocked the antidepressant-like effects of both arketamine and esketamine [48]. Furthermore, arketamine produced more potent beneficial effects than esketamine on ameliorating the reduced BDNF–TrkB signaling observed in the PFC and hippocampus of CSDS-susceptible mice [48]. Moreover, the regulation of astrocytic glutamate transporter 1 by TrkB signaling plays a role in the antidepressant-like effects of (*R,S*)-ketamine in a chronic unpredictable mild stress model [117]. We previously demonstrated that mice lacking the transcription factor *Nrf2* (*Nrf2*-KO mice) show a depression-like phenotype through decreased BDNF–TrkB signaling in the PFC and hippocampus [118]. Recently, we reported that arketamine showed rapid-acting and sustained antidepressant-like effects in *Nrf2*-KO mice through TrkB activation [119]. Taken together, these studies suggest that activation of the BDNF–TrkB cascade in the PFC and hippocampus might be implicated in the long-lasting antidepressant effects of (*R,S*)-ketamine and its enantiomers [41–44].

Using conditional *Bdnf*-KO mice, Anderzhanova et al. [120] reported that injection of esketamine (10 or 50 mg/kg) stimulated extracellular levels of mature BDNF in the medial PFC, in an FK-506- binding protein 51-dependent manner. Therefore, it would be interesting to determine the levels of mature BDNF in the medial PFC after injection of arketamine. Furthermore, D-serine, an endogenous co-agonist at NMDAR, showed antidepressant-like effects in control rats through AMPAR activation and subsequently increased BDNF expression in rat hippocampus [121]. A recent gene-based genome-wide association study in Taiwan showed the predictive role of BDNF–TrkB signaling, glutamatergic signaling, and GABAergic signaling in the antidepressant actions of (*R,S*)-ketamine in patients with treatment-resistant MDD [122]. Although BDNF plays an important role in antidepressant-like effects of ketamine and its two enantiomers, the precise molecular mechanisms underlying ketamine’s actions on BDNF–TrkB signaling remain poorly understood.

In 2021, Casarotto et al. [123] reported that all antidepressants (i.e., imipramine, fluoxetine, venlafaxine, moclobemide, (*R,S*)-ketamine, and esketamine) bind to the transmembrane domain of TrkB. Furthermore, the antidepressant candidate (2 *R*,6 *R*)-HNK, but not its enantiomer (2 *S*,6 *S*)-HNK, binds to the transmembrane domain of TrkB, although it should be noted that (2 *S*,6 *S*)-HNK, but not (2 *R*,6 *R*)-HNK, showed antidepressant-like effects in chronic corticosterone-treated mice [70]. Casarotto et al. concluded that the binding of all antidepressants, including (*R,S*)-ketamine, esketamine, and (2 *R*,6 *R*)-HNK, to the transmembrane domain of TrkB, is the common mechanism of antidepressant effects. However, there are several concerns regarding this paper. First, the effects of arketamine were not investigated in the same assays, despite arketamine showing more potent antidepressant-like effects than esketamine and (2 *R*,6 *R*)-HNK in rodents. Second, only the forced swimming test was performed in TrkB^WT^ and TrkB^Y433F^ mice without a depression-like phenotype in order to investigate antidepressant-like actions. Further study using several behavioral tests and rodents with depression-like phenotypes is required to confirm the role of TrkB^Y433F^ in antidepressant-like actions. In addition, the inhibition constant of (*R,S*)-ketamine for TrkB (Ki = 12.30 μM) is less potent than that of esketamine (Ki = 2.86 μM) and (2 *R*,6 *R*)-HNK (Ki = 2.23 μM), which is inconsistent with their respective potencies for antidepressant-like effects in rodents. Finally, it is known that (*R,S*)-ketamine can elicit robust antidepressant actions in patients with treatment-resistant MDD or BD who did not respond adequately to two or more courses of other current antidepressants. Thus, the conclusion of the article by Casarotto et al. [123] is contradictory to the clinical evidence from (*R,S*)-ketamine in patients with treatment-resistant MDD. Therefore, further detailed study is needed to confirm the hypothesis that binding to the TrkB transmembrane domain is a mechanism common to all antidepressants.

#### Transforming growth factor β1(TGF-β1) system

As we have discussed here, arketamine has more potent antidepressant-like effects than esketamine in rodents; however, the precise molecular mechanisms underlying the differences between the two enantiomers remain unclear. Using RNA sequencing in the PFC of a CSDS model, we found that transforming growth factor β1 (TGF-β1) plays a role in the antidepressant-like effects of arketamine [124]. TGF-β1 and its receptors are expressed in microglia, but not astrocytes, in the mouse PFC. Interestingly, partial depletion of microglia in the PFC by PLX3397, an inhibitor of colony-stimulating factor 1 receptor (CSF1R), blocked the antidepressant-like effects of arketamine in a CSDS model, suggesting a potential role for microglial TGF-β1 in the antidepressant-like effects of arketamine [124]. Furthermore, intracerebroventricular injection of recombinant TGF-β1 resulted in rapid and sustained antidepressant-like effects in a CSDS model [124]. Moreover, we found that intranasal administration of TGF-β1 elicited rapid-acting and sustained antidepressant-like effects in a CSDS model, and that the TrkB antagonist ANA-12 blocked these TGF-β1 effects [125], suggesting a role of TrkB signaling in the antidepressant-like effects of TGF-β1.

**Fig. 2:**
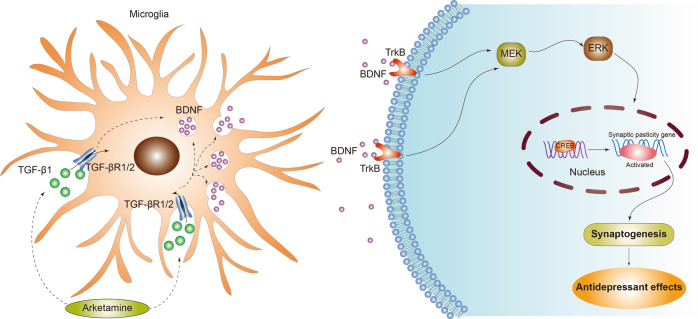
Proposed signaling pathways underlying the antidepressant-like actions of arketamine and TGF-β1. Arketamine induces the expression of TGF-β1 in the microglia through unidentified mechanisms. Arketamine-induced TGF-β1 or TGF-β1 bind to its receptor TGF-β receptor 1/2 in　the microglia. Subsequently, released BDNF binds to its receptor TrkB, resulting in MEK–ERK–CREB signaling pathway, leading to synaptogenesis and antidepressant actions. MEK: mitogen-activated protein kinase, ERK: extracellular signal-regulated kinase, CREB: cAMP response element binding protein.

Chronic stress has been shown to cause alterations in BDNF and TGF-β1 levels in different brain regions of rodents [126–128]. Interestingly, Sometani et al. [129] reported that TGF-β1 potentiated BDNF expression in cultured cerebral cortex neurons, partly via the neurotrophic activity of TGF-β1. Interestingly, alterations in the TGF-β1 system have been reported in MDD patients [130–133]. Given the evident interplay between the BDNF–TrkB and TGF-β1 systems, we suggest that the BDNF–TrkB system plays a role in the rapid-acting antidepressant-like actions of TGF-β1 (Fig. 2), and although further study of this interaction is needed, intranasal TGF-β1 administration could be a novel therapeutic approach for depression.

### mTORC1, extracellular signal-regulated kinase, eEF2K, 4E-BPs, and neuregulin-1

In 2010, Li et al. [102] demonstrated that rapamycin, a mechanistic target of rapamycin (mTOR) inhibitor, blocked the antidepressant-like effects of (*R,S*)-ketamine in rodents, implicating mTOR complex 1 (mTORC1) as a mediator of (*R,S*)-ketamine’s antidepressant-like effects. It has been shown that mTORC1 plays a role in the antidepressant-like effects of esketamine, but may not play such a role for arketamine [51]. Rather, the antidepressant-like actions of arketamine may be mediated by extracellular signal-regulated kinase (ERK) activation (Fig. 2) [51]. Unexpectedly, pretreatment with rapamycin did not block, but rather prolonged, the antidepressant actions of (*R,S*)-ketamine (0.5 mg/kg) in patients with treatment-resistant MDD [134]. At present, studies into the role of mTORC1 in the antidepressant-like effects of (*R,S*)-ketamine and its enantiomers have produced inconsistent results [41–44]. Further study is needed to fully understand the role of mTORC1 signaling in the actions of (*R,S*)-ketamine and its enantiomers.

Eukaryotic elongation factor 2 kinase (eEF2K) is a protein kinase that regulates the elongation stage of protein synthesis. Monteggia et al. [135] proposed that eEF2K plays a role in mediating the antidepressant-like effects of (*R,S*)-ketamine, which did not show antidepressant-like effects in *Eef2k* KO mice; however, it is noted that these mice did not display a depression-like phenotype [136]. It is known that mTORC1 controls several functions through translational regulation by eukaryotic initiation factor 4E-binding proteins (4E-BPs). In 2021, Aguilar-Valles et al. [137] reported that 4E-BPs play a role in the antidepressant-like effects of (*R,S*)-ketamine and (2 *R*,6 *R*)-HNK, although they did not use mice with depression-like behaviors. Further studies in rodents with depression-like phenotypes are needed to ascertain the role of eEF2K and 4E-BPs in the antidepressant-like effects of (*R,S*)-ketamine and its metabolites.

Ketamine is known to modulate the excitatory and inhibitory balance in the PFC. The epidermal growth factor family member neuregulin-1 (NRG1) and its receptor ErbB4 play a role in the regulation of inhibitory neural circuits in the PFC [138]. A recent study showed that the rapid and sustained antidepressant-like effects of (*R,S*)-ketamine may be mediated through cortical disinhibition via PV-specific NRG1 signaling in the medial PFC [139]. Future studies using rodents with depression-like phenotypes are also required to confirm the role of NRG1 in the antidepressant-like effects of (*R,S*)-ketamine and its enantiomers.

### Brain–body crosstalk

#### Brain–gut–microbiota axis

The gut microbiome and its associated short-chain fatty acids (SCFAs) play a role in the brain–gut–microbiota axis, which is involved in psychiatric and neurological disorders [140–145]. We reported that abnormalities in the composition of gut microbiota and SCFAs may contribute to resilience versus susceptibility in rodents exposed to stress [146–149]. Furthermore, the brain–gut–microbiota axis acts via the vagus nerve to play a key role in depression-like phenotypes in mice after transplantation of “depression-related microbes” [150, 151]. Interestingly, (*R,S*)-ketamine and arketamine ameliorated the abnormal gut microbiota composition in mice with a depression-like phenotype [152–155]. Thus, the brain–gut–microbiota axis may, at least in part, play a role in antidepressant-like actions of (*R,S*)-ketamine and arketamine, although further studies are needed to confirm the involvement of this axis [156].

#### Brain–spleen axis

**Fig. 3:**
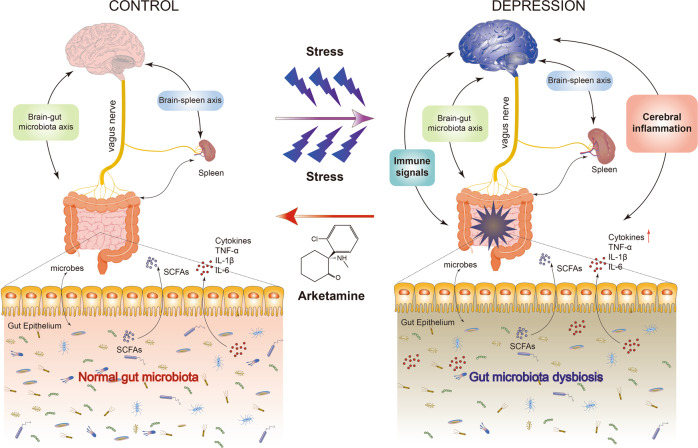
Role of brain–gut–microbiota axis and brain–spleen axis in the stress-related psychiatric disorders and beneficial effects by arketamine. Repeated stress caused gut microbiota dysbiosis and an increase in spleen size and weight, resulting in abnormalities in immune system. Stress-induced neuroinflammation might be mediated by the brain–gut–microbiota axis and the brain–spleen axis through the vagus nerve. Interestingly, arketamine could ameliorate the abnormalities of gut microbiota, abnormal functions of the spleen, and depressive symptoms in patients with stress-related disorders. A slight modification from the previous report [44]. Some materials of the figure have been designed using resources from Freepik.com.

The spleen is a large immune organ that plays a key role in the regulation of erythrocytes and the immune system. We recently reported an abnormality in the composition of mouse gut microbiota and SCFAs after splenectomy, suggesting a role for spleen in the brain–gut–microbiota axis [157]. CSDS-susceptible mice showed a larger spleen volume than in control (no CSDS) and CSDS-resilient mice[158]; interestingly, a single injection of arketamine could ameliorate the increased splenic weight in CSDS-susceptible mice [158]. Depression-like behaviors, increased spleen volume, and abnormal composition of gut microbiota following injection of lipopolysaccharide (LPS) in mice were attenuated after subdiaphragmatic vagotomy, further suggesting a role of the brain–gut–microbiota axis via the vagus nerve [159]. Splenectomy prior to chronic sleep restriction abrogated the enhancement of LPS-induced increases in neuroinflammation and abnormal cognition and anxiety behaviors, implicating the spleen in sleep restriction-induced exacerbation of LPS-induced brain damage [160]. Moreover, stress-activated corticotropin-releasing hormone neurons control adaptive immunity in the spleen by direct innervation, suggesting a brain–spleen axis in the regulation of humoral immunity [161, 162]. Collectively, it is likely that brain–spleen axis and brain–gut–microbiota axis via the vagus nerve play crucial roles in stress-related disorders (Fig. 3). In addition, using postmortem tissues, we reported correlations between BDNF propeptide in the brain and spleen [163], and a negative correlation between CSF1R and transcription factor PU.1 (SPI1) [164], suggesting a role for the brain–spleen axis in psychiatric disorders such as depression. It will be interesting to examine whether arketamine influences the observed abnormalities in the brain–spleen axis in stress-related psychiatric disorders (Fig. 3).

### Beyond depression

#### Cognitive impairments in psychiatric disorders

Cognitive impairments have been shown in a range of psychiatric disorders, including schizophrenia, MDD, BD, PTSD, general anxiety disorder, obsessive compulsive disorder, autism spectrum disorder, attention-deficit hyperactivity disorder, and panic disorder [165]. In addition to positive and negative symptoms, intravenous administration of (*R,S*)-ketamine (0.5 mg/kg) was reported to produce cognitive impairments in healthy control subjects [166]. A recent randomized, double-blind, placebo-controlled study in young healthy subjects demonstrated that intravenous administration of esketamine (0.1 mg/kg/min for 5 min and 0.006 mg/kg/min for 60 min) and (*R,S*)-ketamine (0.2 mg/kg/min for 5 min and 0.012 mg/kg/min for 60 min) produced significant psychopathological and neurocognitive impairment compared to the placebo [167]. Interestingly, esketamine, but not (*R,S*)-ketamine, significantly increased the auditory alterations subscore of the five-dimensional questionnaire for the assessment of altered states of consciousness; this finding suggests that arketamine exerts a potential protective effect against esketamine-induced psychotomimetic effects [167].

Surprisingly, six infusions of (*R,S*)-ketamine (0.5 mg/kg) significantly ameliorated cognitive impairment, as measured by processing speed, in patients with treatment-resistant MDD or BD [168–170]. A recent systematic review revealed that (*R,S*)-ketamine infusion showed significant improvements in cognitive impairment in patients with treatment-resistant MDD, although (*R,S*)-ketamine did not worsen cognitive function in depressed patients [171], as had been observed in healthy controls [166, 167]. Furthermore, it was suggested that the improvement in working memory may be predictive of the anti-suicidal-ideation response to (*R,S*)-ketamine in patients with treatment-resistant MDD [172]. Thus, it is noteworthy that (*R,S*)-ketamine has beneficial effects on cognitive impairment in depressed patients.

**Fig. 4:**
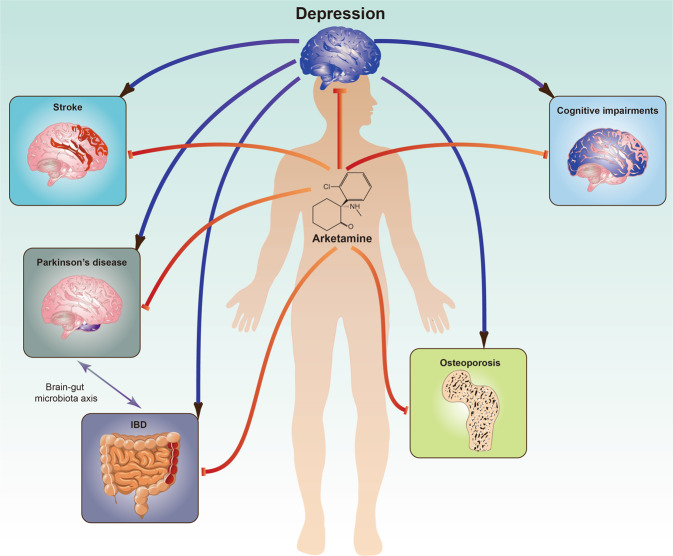
Potential of arketamine for cognitive impairments in psychiatric disorders and other diseases. Preclinical findings suggest that arketamine would be a new therapeutic drug for cognitive impairments in psychiatric disorders, neurodegenerative disorders such as Parkinson’s disease, osteoporosis, IBDs (ulcerative colitis and Crohn’s disease), and stroke. Importantly, patients with these diseases have depressive symptoms as comorbidity. IBD is a risk factor for PD [180]. Some materials of the figure have been designed using resources from Freepik.com.

In addition, we reported that PCP-induced cognitive deficits in mice were ameliorated after subsequent repeated intermittent administration of arketamine, but not esketamine, and that these effects were mediated by BDNF–TrkB activation [173]. As cognitive impairment can influence quality of life, it will be important to investigate whether arketamine can improve cognitive impairment in patients with psychiatric disorders (Fig. 4).

#### Parkinson’s disease

Parkinson’s disease (PD) is a chronic and progressive neurodegenerative disorder, in which depression is common [174]. In a mouse model of PD, 1-methyl-4-phenyl-1,2,3,6-tetrahydropyridine (MPTP)-induced dopaminergic neurotoxicity in the mouse striatum was attenuated by treatment with repeated intranasal administration of arketamine, but not esketamine [175]. Furthermore, pretreatment with the TrkB inhibitor ANA-12 significantly blocked the beneficial effects of arketamine against MPTP neurotoxicity, suggesting that TrkB plays a role in the protective effects of arketamine. Therefore, arketamine could be a potential novel therapeutic for the treatment of neurodegenerative disorders such as PD (Fig. 4).

#### Osteoporosis

Depression is common in, and is a risk factor for, osteoporosis, particularly in women. Kadriu et al. [176] reported that bone inflammation markers might be involved in the antidepressant actions of (*R,S*)-ketamine in patients with treatment-resistant MDD. Arketamine, but not esketamine, significantly attenuated the increased plasma levels of receptor activator of nuclear factor κB ligand (RANKL) observed in CSDS-susceptible mice [177]. Interestingly, there was a positive correlation between sucrose preference and ratio of osteoprotegerin (OPG) and RANKL [177]. Arketamine, but not (2 *R*,6 *R*)-HNK, ameliorated the increased plasma levels of RANKL and decreased OPG/RANKL ratio found in CSDS-susceptible mice. Moreover, arketamine, but not its metabolite (2 *R*,6 *R*)-HNK, ameliorated the decreased bone mineral density in CSDS-susceptible mice [178]. The decreased bone mineral density observed in ovariectomized mice was also ameliorated after subsequent repeated intermittent administration of arketamine, but not esketamine [179]. These findings all suggest that arketamine could potentially be used as a therapeutic treatment for bone metabolism abnormalities in patients with MDD or osteoporosis (Fig. 4).

#### Inflammatory bowel diseases

Ulcerative colitis (UC) is a chronic inflammatory bowel disease (IBD) that causes long-lasting inflammation, ulcers, and colitis in the gastrointestinal tract. Depression is a common symptom in patients with UC, and is itself a risk factor for IBDs [180]. Accumulating evidence suggests that IBD might increase the risk of PD through the brain–gut–microbiota axis [181]. In a dextran sulfate sodium (DSS)-induced mouse model of colitis, repeated administration of arketamine, but not esketamine, significantly ameliorated the DSS-induced inflammation and colitis through TrkB activation [182]. These data suggest that arketamine could be a potential therapeutic drug for IBD (Fig. 4). Therefore, a further double-blind, placebo-controlled study of arketamine in IBD patients with or without depression would be of much interest.

#### Stroke

Stroke is the most common acute cerebrovascular disease. Importantly, poststroke depression occurs in a number of patients with stroke, leading to greater disability as well as increased mortality [183]. Brain injury and behavioral abnormalities in mice after middle cerebral artery occlusion (MCAO) were attenuated by subsequent administration (1 and 24 h after MCAO) of arketamine but not of esketamine [184]. This study suggests that arketamine may have potential as a new therapeutic drug for ischemic stroke and poststroke depression (Fig. 4).

## Conclusion

Considering the preclinical findings in studies of the two ketamine enantiomers, and the inability of non-ketamine NMDAR compounds to replicate the effects of (*R,S*)-ketamine in MDD patients, it is unlikely that NMDAR plays a major role in the antidepressant effects of (*R,S*)-ketamine and arketamine. However, further study in this field is required. At present, the primary molecular mechanism by which arketamine exerts its antidepressant actions is unknown. Further study using new technologies such as chemical biology is needed to fully understand the molecular pathways of arketamine and identify novel targets for treatment intervention.

An open-label study of arketamine in patients with treatment-resistant MDD showed robust antidepressant actions [87]; these results must be confirmed in a randomized, placebo-controlled, double-blind study using a large sample size. A clinical trial of arketamine by Perception Neuroscience, Inc. (New York, USA) is underway. In February 19, 2021, Perception Neuroscience announced the positive data from the first Phase 1 study showing the safety and tolerability of arketamine [185]. Arketamine was safe and well-tolerated at all doses up to 150 mg. Furthermore, arketamine required substantially higher doses to induce similar perceptional changes than esketamine [185]. Phase 2 study of arketamine in patients with treatment-resistant depression will be started from the second quarter of 2021.

A future randomized, double-blind study of arketamine versus esketamine [or (2 *R*,6 *R*)-HNK] in patients with treatment-resistant MDD is needed to ascertain the role of the NMDAR in the robust antidepressant actions of (*R,S*)-ketamine. Finally, we propose that arketamine could prove beneficial as a treatment for MDD, BD, PTSD, cognitive impairment in psychiatric disorders, PD, osteoporosis, IBD, and stroke.
